# Survive or thrive after ICU: what’s the score?

**DOI:** 10.1186/s13613-023-01140-x

**Published:** 2023-05-19

**Authors:** Bairbre A. McNicholas, Ryan Haines, Marlies Ostermann

**Affiliations:** 1 Department of Anaesthesia and Intensive Care Medicine, School of Medicine, University of Galway, Galway, H91 YR71 Ireland; 2 Adult Critical Care Unit, The Royal London Hospital, Barts Health NHS Trust, London, UK; 3 William Harvey Research Institute, Queen Mary University of London, London, UK; 4 Department of Intensive Care, King’s College London, Guy’s and St Thomas’ Hospital, London, UK

AKI is a frequent complication in critically ill patients and portends poor short and long-term outcomes, including an increase in readmissions, cardiovascular events, and progression to chronic kidney disease (CKD) [1]. The impact on health related quality of life (HRQOL) has been studied less. Further, it is not known which aspects of HRQOL are most affected and why, and how this could shape post-critical care management. Some of these questions have been addressed in the follow-up study of The Artificial Kidney Initiation in Kidney Injury (AKIKI) trial by Chaibi et al. [2].

AKIKI was a large French multicentre randomised controlled trial on renal replacement therapy (RRT) initiation strategy for AKI [2]. Chaibi et al. examined longer-term survival, renal outcomes and HRQOL in 316 patients (51% of total population) who survived 60 days after randomization. Their median follow-up was up to 3.35 years [Interquartile range (IQR) 1.89—4.09]. Survival rate was 39.4% at three years following inclusion, with age being the only predictor om mortality. Over a quarter of patients had worsening renal function whilst 5% needed chronic dialysis. HRQOL was assessed using the Eq-5L instrument at a median of 3 years post randomisation. Although the response rate was low at 35%, overall HRQOL following an ICU admission with AKI was low. Population normative data for the EQ-index is generally 0.80–0.85 [3] and the median index value in this study was 0.67 (IQR 0.40 to 1.00). Interestingly, HRQOL scores were not influenced by need for chronic dialysis.

These findings are consistent with a body of literature that was summarized in a comprehensive meta-analysis published in 2014 [3]. The meta-analysis included 18 studies over a 18 year period in which six different HRQOL assessment tools (SF-46, EQ-5L, NHP, HUI3, MOS-SF-20, SF-12) were used and assessments were made over a median of 10.5 months ranging from 2 to 14 years after ICU admission [3]. Overall, HRQOL was markedly impaired among survivors of AKI compared to the general population and this was mainly driven by limitations in physical function, mobility and ambulation compared to psychosocial domains. Interestingly, the majority of studies found a similar degree of reduction in HRQOL in patients experiencing AKI or receiving RRT when compared to similarly critically ill patients without severe AKI. Notably, at 1 year post ICU admission, more than 80% of respondents would undergo ICU admission again if needed to survive [4].

Since that publication, several other studies have examined HRQOL outcomes with similar findings (Table 1). Further, additional scoring systems have been introduced, notably the clinical frailty score (CFS). Frailty, although more common with age but not confined to the elderly, complicates acute and chronic disease and is considered a marker of a limited existence [5]. It can be assessed using the CFS, a validated measure of clinical frailty with scores ranging from 1 (very fit) to 7 (severely dependent). There are recognized limits to the CFS, and although not a classic HRQOL scoring system, it is objective, easy to obtain and easy to teach [6]. In a prospective multicentre observational study enrolling older critically ill patients with AKI, frailty was defined as having a CFS > 5. The study found 28% of survivors were considered frail at 6 months, of whom 57% were not back at baseline (pre-ICU level) with only 4% transitioning to not frail and a further 4% becoming frail [5]. A follow-up of the BRAIN ICU study, a prospective cohort of critically ill adults with acute respiratory failure and/or shock enrolled found that severity of AKI was associated with increasing frailty at both 3 and 12 months compared to baseline measurements [6]. These studies further corroborate the ongoing decline in physical function of patients who suffer AKI as part of their critical illness. They also highlight the link between frailty and HRQOL and the complexity of assessing HRQOL specifically related to survival after AKI.

**Table 1:** Selected studies exploring quality of life and frailty in AKI survivors

Study	Location	Years of enrolment	Design	AKI population	QOL or Frailty questionnaire Response rate / total population	Instrument	Assessment time point	Comments/Main findings
Soliman et al. [7]	Netherlands	July 2009 until April 2013	Single center retrospective analysis	Patients with early RIFLE AKI in ICU and alive at 1 year	1020 / 1549 (65.8%)	EuroQoL 5D-3L™ (EQ-5D)	1 year	All AKI categories were associated with a primary outcome of EQ-5L < 0.4 or death but at 1 year, AKI category was not associated with HRQOL
Salathe et al. [9]	Switzerland	January 2015 until April 2018	Single site observational study	All patients > 55 who received RRT for AKI and were alive in May 2019	83/119 (69.7%)	EQ-5D with VAS		VAS score was 71 (SD 22) and mean EQ-5D derived health utility 0.76 (IQR 0.26). Pain was the most frequently reported limitation (46.9%), followed by mobility (36.1%) and anxiety (21.6%); Scores were significantly lower in patients older than 75 years compared to younger patients; QOL was significantly lower than an age/sex matched reference population
Thanapongsatorn et al. [10]	Thailand	August 2018 to January 2021	Randomised controlled trial	Severe AKI stage 2–3 and alive at 12 months	78/98 (79%)	EQ-5D-5L	1 year	No statistically significant difference in EQ-5D-5L index scores between the comprehensive care and control group (0.99 [0.8–1.0] vs 0.96 [0.8–1.0], p = 0.80)
Mishra et al. [11]	United Kingdom	January 2005 until December 2011	Observational cohort study	Patients with RIFLE –I or higher AKI post cardiac surgery and alive at least 1 year post surgery	499/777 (64%)	SF-12 v2)	At least 1 year	Median follow-up for patients who returned the QOL questionnaires were 60 months (30, 113) for the AKI group and 63 months (30, 112) for non-AKI patients. Mental scores were not significantly different (51.0 [18.5, 70.8] vs. 52.2 [21.8, 70.6], *P* = 0.2) between both groups but the physical scores were (38.8 [14.2, 62.5] vs. 44.2 [13.8, 76.7], *P*< 0.01)
**Studies examining clinical frailty score post AKI**								
Beaubien-Souligny et al. [5]	Canada	September 2013 until November 2015	Multicentre prospective cohort study at 6 and 12 months	ICU patients ≥ 65 years with severe AKI and alive at 90 days	243 / 499 at 6 months (87%) 216 / 499 at 12 months (81%)	CFS	At baseline,90 days and 6 months	Frailty was independently associated with 90-day mortality (adjusted HR 1.49; 95% CI 1.11–2.01, *p* = 0.008); 243 (53%) patients were alive and had CFS scores captured. Among these, 68 (28%) were frail including 39 (57%) patients who were not frail at baseline
Abdel-Kader et al. [6]	USA	2007 to 2010	Prospective cohort study in 5 US medical centers	Critically ill adults with acute respiratory failure and/or septic or cardiogenic shock and KDGIO-AKI	317/371 at 3 months (85%) 318/371 at 12 months (86%)	CFS	Baseline 3 and 12 months	Peak AKI was generally associated with higher CFS scores at 12 months (AKI stage 1: OR 1.87, 95% CI 1.11, 3.14; AKI stage 2: OR 1.81, 95% CI 0.94, 3.48; AKI stage 3: OR 2.76, 95% CI 1.34, 5.66)

*AKI* acute kidney injury, *HR* hazard ratio, *RIFLE* Risk–Injury–Failure–Loss–End-stage, *RRT* renal replacement therapy, *VAS* visual analog scale, *IQR* interquartile range, *CFS* clinical frailty score, *QOL* quality of life, *KDIGO* Kidney Disease Improving Global Outcomes, *OR* odds ratio, *CI* Confidence interval

There are additional limitations to interpreting HRQOL data following critical illness. First, the findings are impacted by the heterogeneity in study design and participants, timing of assessment, and tools used. Second, most studies reported a high loss to follow-up [3]. Despite this, there are useful signals, including the absence of difference in HRQOL based on stage of AKI or implementation of acute RRT [3, 6, 7]. This suggests that the process that led to AKI drives ongoing worse outcomes rather than AKI per se. More recently, worse cardiovascular outcomes have been reported for patients who survive AKI during hospitalisation, particularly in those with increased proteinuria [1]. Measuring and monitoring such outcomes provide valuable data of the long-term effects of AKI. However, the day to day effects long after ICU admission are less well studied, nor how the effects of changing socio-economic status following critical care influence HRQOL measurements at the time of testing [8].

The study by Chaibi and colleagues provides important data to our field on how patients survive after critical illness. However, it is unclear how these results should influence clinical management. Should rehabilitation, optimisation of nutrition and psychosocial support to reduce frailty be as much part of follow-up care after AKI as measuring urinary albumin and serum creatinine? The study by Chaibi et al. serves as a reminder that more research in AKI survivors is urgently needed to inform management strategies so that patients with AKI do not just survive but thrive after ICU.

## Data Availability

Not applicable.

## References

[1] Hsu CY, Chinchilli VM, Coca S (2020). Post-acute kidney injury proteinuria and subsequent kidney disease progression: the assessment, serial evaluation, and subsequent sequelae in acute kidney injury (ASSESS-AKI) study. JAMA Intern Med.

[2] Chaibi K, Ehooman F, Pons B (2023). Long-term outcomes after severe acute kidney injury in critically ill patients: the SALTO study. Ann Intensive Care.

[3] Villeneuve PM, Clark EG, Sikora L, Sood MM, Bagshaw SM (2016). Health-related quality-of-life among survivors of acute kidney injury in the intensive care unit: a systematic review. Intensive Care Med.

[4] Oeyen S, De Corte W, Benoit D (2015). Long-term quality of life in critically ill patients with acute kidney injury treated with renal replacement therapy: a matched cohort study. Crit Care.

[5] Beaubien-Souligny W, Yang A, Lebovic G, Wald R, Bagshaw SM (2021). Frailty status among older critically ill patients with severe acute kidney injury. Crit Care.

[6] Abdel-Kader K, Girard TD, Brummel NE (2018). Acute kidney injury and subsequent frailty status in survivors of critical illness: a secondary analysis. Crit Care Med.

[7] Soliman IW, Frencken JF, Peelen LM (2016). The predictive value of early acute kidney injury for long-term survival and quality of life of critically ill patients. Crit Care.

[8] Falvey JR, Murphy TE, Leo-Summers L, Gill TM, Ferrante LE (2022). Neighborhood socioeconomic disadvantage and disability after critical illness. Crit Care Med.

[9] Salathe C, Poli E, Altarelli M, Bianchi NA, Schneider AG (2021). Epidemiology and outcomes of elderly patients requiring renal replacement therapy in the intensive care unit: an observational study. BMC Nephrol.

[10] Thanapongsatorn P, Chaikomon K, Lumlertgul N (2021). Comprehensive versus standard care in post-severe acute kidney injury survivors, a randomized controlled trial. Crit Care.

[11] Mishra PK, Luckraz H, Nandi J (2018). Long-term quality of life postacute kidney injury in cardiac surgery patients. Ann Card Anaesth.

